# A Combined Tie-Fastening Method for the Reusable Surgical Gown with Two Neck Tie Belts to Improve Wearing Comfort

**DOI:** 10.3390/nursrep10020011

**Published:** 2020-11-16

**Authors:** Kai-Hui Chang, Yu-Ling Chen, Shu-Yi Dai

**Affiliations:** Nursing Department, Chiayi Christian Hospital, Chiayi 60002, Taiwan; 04642@cych.org.tw (K.-H.C.); 02035@cych.org.tw (Y.-L.C.)

**Keywords:** combined tie-fastening, neck tie, reusable, surgical gown, X-ray protective apron

## Abstract

The reusable surgical gowns made of slippery materials have the tendency to slip down as they are being worn. The rear neck tie(s) can sometimes loosen, and this causes the surgical gown to slip down somewhat, making the surgical staff members feel uncomfortable. If the gowns have two rear neck ties with a tendency of loosening and allowing the gowns to slip down, the surgical staff members feel more uncomfortable when there is only one tie loosening but the other tie is tethering. To fasten the neck ties of the surgical gown with two neck tie belts, we propose a simplified method of fastening the two sets of tie belts together as one tie, instead of fastening them separately. The object of this study is to evaluate this combined tying method for its ability to secure the gown and its wearing comfort. We enrolled five volunteers to evaluate the tie loosening condition of the reusable surgical gowns with two sets of rear neck tie belts after a series of upper limb motion exercises while wearing and not wearing the X-ray protective apron beneath the gown. The amount of uppermost rear neck cloth edge separation was recorded before and after the exercise. We also evaluated the wearing comfort of five enrolled operating surgeons for comparing the original and this modified tying method while wearing and not wearing the X-ray protective apron. In the results, we found that combined tying tends to have significantly more rear gown separation (0.94 cm) than separate tying (0.27 cm) after vigorous upper extremities exercise. However, during the actual performance of the surgeries, the rear neck tie(s) loosening and wearing discomfort of the combined tying method was significantly less than using the separate tying method (loosening: 0% vs. 30%) (discomfort: 0% vs. 35%) while the X-ray protective apron was not worn. For reusable surgical gowns that have two rear neck ties, we suggest the two sets of rear neck ties could be fastened together as one combined tie in routine surgical practice. With this, tying could be performed easier and faster, and wearing comfort could be improved.

## 1. Introduction

The wearing of a sterile surgical gown by perioperative specialists prior to and during major surgeries is an important routine and a critical technique [1,2,3,4] to prevent surgical site infection [5,6,7]. For this purpose, use of a disposable surgical gown is convenient and popular. However, many institutions still mainly use reusable surgical gowns for their surgeries for various reasons [8,9]. Waterproof or water-resistant material is one of the materials commonly used for reusable surgical gowns. These cloth materials give the gown a smooth surface, which makes them slippery. So, when someone wears a gown made of a slippery cloth, that gown has a tendency to slip along or down from his/her shoulders.

**Figure 1:**
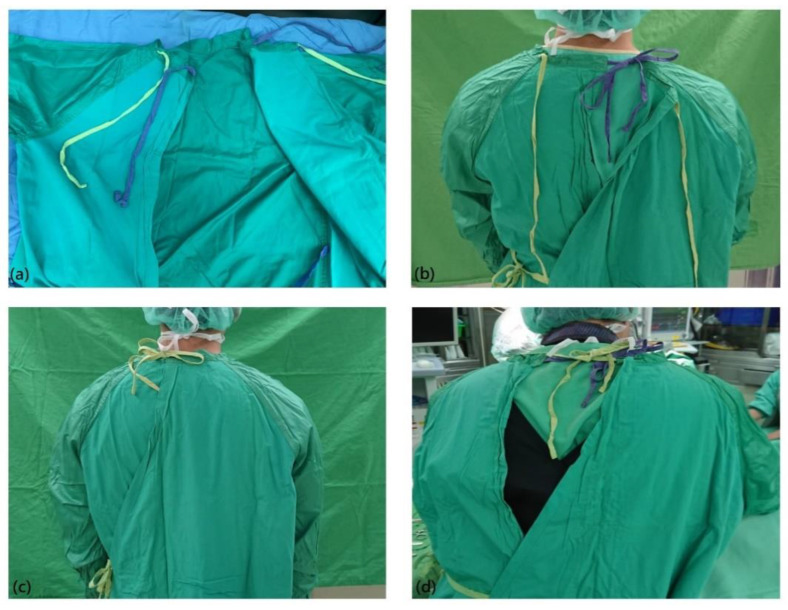
The photographs illustrate (**a**) the design of two sets of rear neck ties, (**b**) the inner tie fastened, (**c**) both ties fastened, (**d**) the ties loosening during surgery.

The rear neck tie(s) and rear waist tie of the reusable gown are designed as belts to be fastened, to allow the gown to wrap around the body, mainly the upper limbs and anterior chest, for a sterile outer surface. The tie belts should be fastened with the aid of a circulating nurse. The rear waist belt, at the waist level, is used mainly to enwrap the surgical staff member. The rear neck tie(s) also can be used to enwrap the body, as they are often designed as inner and outer ties on the reusable gown (Figure 1). In addition, they play an important role in keeping the gown from slipping down. Surgical gown contamination is a critical issue that could lead to surgical site infection, and cause the failure of the surgery. If the gown slipping down somewhat, the key sterile part will not be maintained, and it would possibly lead to an increased infection rate [10,11,12].

Surgical gowns made of slippery materials have the tendency to slip down due to gravity and physical movements as they are being worn. The rear neck tie(s) can sometimes loosen (Figure 1), and this causes the surgical gown to slip down more, making the surgical staff members feel uncomfortable. The discomfort becomes worse when there is only one tie loosening and the other tie tethering. The increased discomfort from the surgical gown would possibly have negative effects on the surgical staff by affecting memory, reaction time, and complex motor skills [13,14,15].

**Figure 2:**
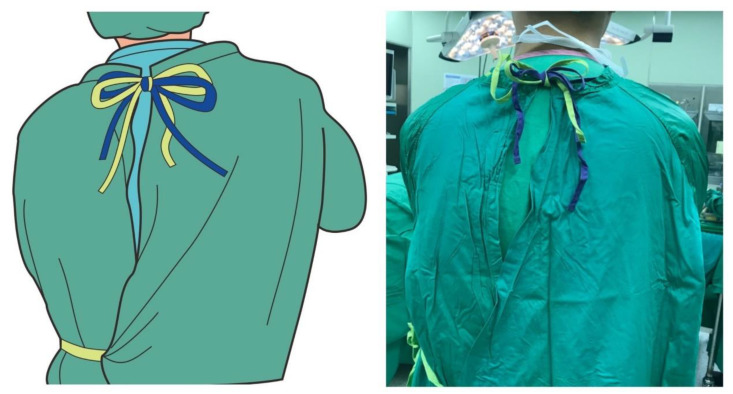
Illustration of the combined tie-fastening method for two sets of rear neck ties.

Surgical staff members at our institute who wear slippery water-resistant reusable surgical gowns often complain that the rear neck ties loosen and the surgical gown slips down. To fasten the neck ties of the surgical gown with two neck tie belts, some circulating nurses in our institute have adopted a simplified method of fastening the two sets of tie belts together as one tie, instead of fastening them separately, in order to facilitate the wearing procedure and avoid the ties loosening (Figure 2). This method mimics the tying of a surgical gown with only one set of rear neck tie belts and it seems that the complaints of ties loosening or wearing discomfort could be decreased. In this study, we evaluated this combined tying method for its ability to secure the gown and its wearing comfort.

## 2. Materials and Methods

**Figure 3:**
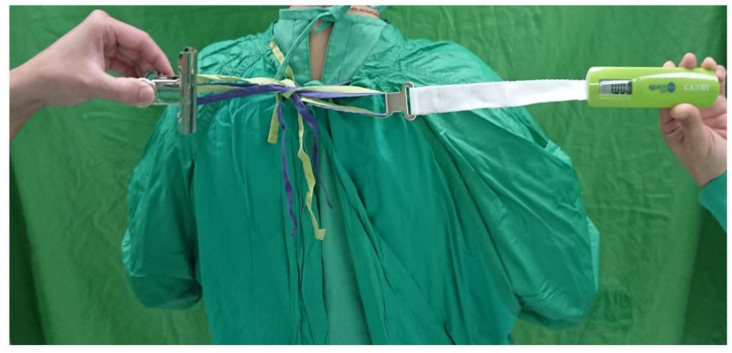
Fastening the ties with a tightening force reaching 1 kg.

**Figure 4:**
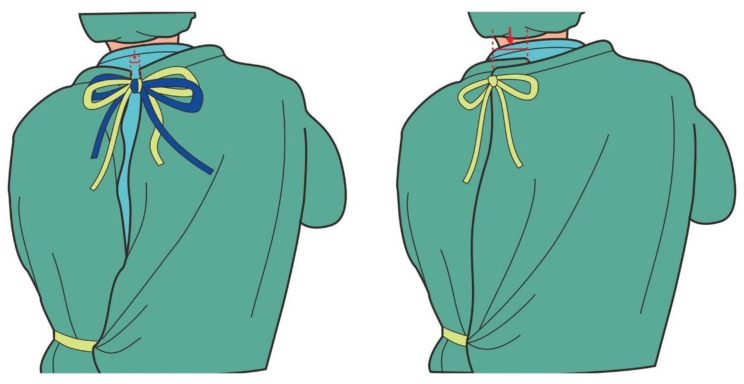
Schematic illustration of the measurement of the rear neck tie(s) loosening by evaluating the separation of the arrow-indicated distance before and after the exercise.

This study was approved by the ethics committee of our institution (IRB number: CYCH-IRB No. 2020021). We conducted a prospective investigation to test the rear neck tie loosening of the separated tie and combined tie methods. The volunteer agreement was signed by all volunteers. We enrolled 5 volunteers (3 males, 2 females) who had at least a 5-year working experience in the operating room with a gown-wearing technique as their routine practice. We aimed to evaluate the tie loosening condition of the reusable surgical gowns with 2 sets of rear neck tie belts after a series of upper limb motion exercises. Each tying method was tested 4 times with all volunteers, and also tested when wearing and not wearing the X-ray protective apron beneath the gown. All the ties were fastened with a tightening force reaching 1 kg, as measured by a weighing device (Figure 3). Then, the volunteers performed 100 rounds of bilateral upper arm horizontal abduction and adduction motions. We evaluated tie loosening after the exercises by evaluating the amount of uppermost rear neck cloth edge separation, which was recorded before and after the exercise (Figure 4).

We also evaluated the wearing comfort of the operating surgeons. Five surgeons were selected for evaluation, and they had different specialties, including hand surgery, foot and ankle surgery, sports arthroscopy, pediatric orthopedics, and orthopedic trauma. A single scrub nurse was involved in the study, but the circulating nurses were not. The scrub nurse (observer) made observations and did the recording. These 2 tying methods could both be seen in our institution. The involved scrub nurse recorded the results by asking the surgeons: 1. Was there any increased wearing discomfort due to the rear neck tie(s) during surgery? 2. Was there any feeling of the rear neck tie loosening at the end of surgery before they took off their surgical gowns? Additionally, any asking for retying of the rear neck ties during surgery or obvious tie loosening at the end of surgery was recorded. Results were recorded as the surgeons performed their specialty surgeries and the results of 4 consecutive times, respectively, of wearing and not wearing the X-ray protective aprons underneath the gown were also recorded for each surgeon.

Data were presented as mean and standard deviation for continuous variables. The chi-square test was used for discrete variables to compare the differences between the 2 groups, and Student’s t-test was used to compare continuous variables. A *p*-value < 0.05 was considered statistically significant. All statistical analyses were performed using statistical software SPSS (IBM SPSS Statistics for Windows Version 24.0, Armonk, NY, USA: IBM Corp, 2016).

## 3. Results

To evaluate surgical gown rear neck ties loosening after exercise, 20 values were obtained for each group with ties separately or together, and with or without wearing an X-ray protective apron inside the surgical gown. The separated distance of the rear neck gown cloth is shown in Table 1. Additionally, the comfort evaluation of the 2 different tying methods is shown in Table 2. All surgeries proceeded following the usual routine and were performed smoothly without interference by the evaluations; there were no perioperative complications.

## 4. Discussion

In this study, we found that combined tying tends to loosen more than separate tying after upper extremities exercise. However, for wearing comfort during the actual performance of the surgeries, the combined tying method was significantly better than the separate tying method. The wearing comfort results were compatible with the observed reality, in that some surgical staff members in our institute changed the separated ties into combined ties to improve wearing comfort in their daily practice. From our results, it would be suggestive that this modified tying method could be adapted into our daily surgical practice.

When the surgical gown is worn, it may slip down if it is not securely wrapped around the body. The surgical staff will feel discomfort when it is slipping halfway down. The rear neck tie(s) of the reusable surgical gown are meant to secure the gown to appropriately fit the surgeon. However, if the tie(s) are not secured, they could loosen over time, especially as the body motions of the staff members wearing the gown increase. Additionally, the slippery material of the surgical gown would more likely lead to ties loosening due to the gown’s tendency to slip down on the staff member’s body.

**Figure 5:**
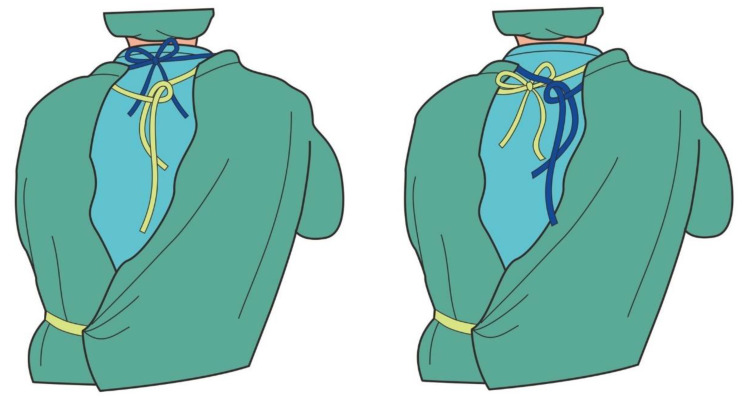
Schematic illustration of the common patterns of rear neck tie loosening.

With the reusable surgical gown with 2 rear neck ties, there is a possible reason for wearing discomfort when the 2 sets of tie belts are tightened under different degrees of tightness in order to pull the gown closer. From the outside, it looks as though the staff member’s back side is well-covered with the surgical gown, but actually the upper part of the gown is secured and supported by only one set of rear neck ties, which is the tighter one. The other set of ties does not contribute to securing and supporting the gown. Discomfort may result from the uneven tightening of the tie belts on the rear of the neck. In addition, the tie may loosen over time, so that the tightness of the tie belts differs more obviously, thereby causing more discomfort (Figure 5).

However, the combined ties distribute the pulling strength equally onto each tie belt, and the staff wearing the gown might feel better than when each tie belt has a different pulling strength. In our after-exercise test, the combined ties showed a higher likelihood of loosening, but wearing comfort was still better than that with separate ties. This may also be because the pulling strength was equally distributed on each rear neck tie belt as they loosened over time; also, body movement during surgery is not as extreme as during our testing exercises, so the tendency to loosen during surgery would be minimized.

Loosening tended to be more significant after exercise while wearing an X-ray protective apron underneath, but for the discomfort evaluation, there was no significant difference after surgery for staff wearing an X-ray protective apron. The main reason for this is that the greater discomfort is related to the wearing of a heavy apron, which tends to minimize the discomfort from the surgical gown. So, this modified tying method would be suitable and practical for the medical staff members who need to wear the X-ray protective apron beneath the gown to perform the sterile work under fluoroscopy.

Another advantage to the combined ties is that they could facilitate wearing and taking off the gowns. The ease in the wearing procedures would be more specific to the newly trained circulating nurse. In other words, when they are handling the 2 neck ties one by one, if the tying is not smooth, he or she will be embarrassed or even afraid of not doing well. So, tying the 2 neck ties together would be a help. Shortening the time of body contact and increasing the ease of performance would allow the newly trained circulating nurse to feel more comfortable and build up confidence.

A limitation of this study is that we tested only one kind of surgical gown, which is used in our institution. No other surgical gowns with different materials were tested. Additionally, the times of evaluations and enrolled cases were not many. The comfort evaluation was performed only for the surgeons and for surgeries of no more than 3 h and no healthcare team was involved, which could also be a weakness of this study. In addition, there are other factors that might contribute to causing the gown to slip down and the comfort question is very subjective and it may be affected by many factors. No use of quantitative values, e.g., Likert scale, for the comfort evaluation is also a limit of this study.

## 5. Conclusions

For the institutes that still use reusable surgical gowns for operations, if the gowns have two rear neck ties with a tendency of loosening and allowing the gowns to slip down, we suggest an alternative method of fastening the two sets of rear neck ties together as one combined set of ties to mimic gowns with only one set of rear neck tie belts. In this way, tying could be performed easier and faster, and wearing comfort could be improved for surgical staffs. However, further comparative studies with more enrolled medical staff members, including healthcare teams, are still needed to determine the general adaptability of this tying method.

## Figures and Tables

**Table 1:** Comparison of tie loosening by measurement of the rear gown cloth separation after exercise.

Measurement	Mean ± SD		*p* Value
	Tying Together (*n* = 20)	Tying Separately (*n* = 20)	
Average rear gown separation (cm)	0.65 ± 0.25	0.49 ± 0.34	0.098
Average rear gown separation, wearing X-ray protective apron inside) (cm)	0.94 ± 0.34	0.27 ± 0.26	<0.001 *

* *p* value < 0.05.

**Table 2:** Surgeons’ responses to different tying methods.

Measurement	Tying Together (*n* = 20)	Tying Separately (*n* = 20)	*p* Value	Tying Together (with Apron) (*n* = 20)	Tying Separately (with Apron) (*n* = 20)	*p* Value
Average operation time (hour)	1.9 ± 1.0	1.8 ± 0.6	0.502	1.8 ± 0.6	1.6 ± 0.5	0.788
Surgeon felt increased discomfort during surgery	0	7	0.008 *	1	3	0.55
Rear neck tie(s) loosening	0	6	0.02 *	3	5	0.27
Surgeon asking for retying during surgery	0	1	1.0	0	0	-

* *p* value < 0.05; apron: X-ray protective apron.
